# Some of the Causes of National Deterioration and Degeneracy*Read before the Texas State Society of Social Hygiene, Augtin, Texas, January 27, 1911.

**Published:** 1912-08

**Authors:** Holman Taylor

**Affiliations:** Fort Worth, Texas. Secretary Texas State Medical Association and Editor of its Journal


### Department of Eugenics.

Conducted by Dr. Malone Duggan and Dr. Theo. Y. Hull.

**THE TEXAS STATE SOCIETY OF SOCIAL HYGIENE.**

Headquarters: San Antonio, Texas.

Dr. Malone Duggan, President, San Antonio.
Dr. F. E. Daniel, 1st Vice President, Austin.
Prof. A. Caswell Ellis, 2d Vice President, Austin.
Mrs Eli Hertzberg, 3d Vice President, San Antonio.
Dr. Theo. Y. Hull, Secretary, San Antonio.
Mr. W. L. Evans, Treasurer, San Antonio.

EXECUTIVE COMMITTEE.

Dr. Malone Duggan, Chm.
Dr. Theo. Y. Hull, Sec'y.
Dr. W. F. McCaleb.
Hon. C. H. Jenkins.
Rev. A. W. S. Garden.
Hon. J. C. Willacy.
Mr. W. L. Evans.
Dr. Frank D. Boyd.
Dr. J. R. Nichols.
Dr. J. M. McCutchan.
Dr. W. A. King.
Dr. J. W. Torbett.
Dr. F. C. Walsh.
Dr. Joe Reuss.
Dr. Frank Paschal.

### Some of the Causes of National Deterioration and Degeneracy.*

BY HOLMAN TAYLOR, B. S., M. D., FORT WORTH, TEXAS.

Secretary Texas State Medical Association and Editor of its Journal.

"To be a good animal is the first requisite to success in life, and to be a nation of good animals is the first condition to national prosperity."

This quotation from the writings of Herbert Spencer has appeared in more than one discussion of this and similar subjects. I know of no better text from which to speak on this occasion.

National history is a constant succession of four epochs: primitive tribal organization, primitive national government, high efficiency civilization and civilization of deterioration. Animal vitality is at its highest point in the first epoch, but begins to deteriorate in the second, because of the temptation to animal excesses produced by closer contact, from which the nation is temporarily saved by the laws and rules of society, adopted as a necessity and taking the place of brute force as a protective measure. The evolution continues step by step to the point of highest efficiency in civilization, where the animal instinct is balanced by reason, and the mind is at its highest point of development. From this point there is a decline, physically, morally and mentally, varying in manner and extent according to the inherent temperament of the people concerned. That the actuating principles in each stage have been fairly constant through all ages can hardly be doubted. One may read them between the lines, as well as in the lines, of all recorded history, even in sacred history. The Biblical history of the Jewish nation illustrates this contention very well. We see the powerful tribes of Israel evolve from the unknown peoples of the earth, assume the scope and power

*Read before the Texas State Society of Social Hygiene, Austin, Texas, January 27, 1911.

of a nation and yield at once to many of the dissipations incident to intimate contact. We see them a wonderful people, strong, valiant and intellectual; then we see them no more as a nation, but in captivity and scattered over the face of the earth. That they have not lost their national characteristics entirely under such adverse circumstances is due, no doubt, to measures adopted in their religion of a sociological and hygienic nature. Greek and Roman civilization, and the conquest of their empires by barbarians, with all that history tells us of their customs and habits, might perhaps be made to track more closely the arbitrary division into epochs I have made here, if time permitted. That there have been conditions of comparative civilization beyond the pale of written history is daily being demonstrated by archaeologists, and that conditions obtained in those days similar in influence on human nature to those existing in more modern times may reasonably be assumed. We may assume, then, that history does repeat itself, and that our boasted civilization with all of its marvelous accomplishments is doomed sooner or later to pass away. That our doom may be postponed to coincide with the end of time is the hope of thoughtful mankind, and this idea is the actuating principle of such gatherings as this of tonight.

The premises laid down by my subject presumes the existence in our nation of a deterioration or degeneration. This presumption is open to attack only on the ground of our continuous progress in educational, social, scientific and religious lines, and because of the great men, great inventions and great fortunes daily produced by existing conditions. In view of the multiplied examples of national greatness one might recite, and because of demonstrable amelioration of previously existed conditions, including an increased longevity, one might well argue that we are not retrogressing as a nation, not even in a single direction. But for the keen observations of sociologists and men of science, backed by incontrovertible statistics gathered at their instance, we would indeed find it a difficult matter to arouse our people from a very comfortable state of lethargy to a disturbing realization of conditions as they actually exist. In contemplating our rapid advancement in the many directions of human interests, we lose sight of the fact that the movement is but a series of processions, led by a few among the many of the rank and file, with the rear of each procession extending far back into the darkness and filth of degradation. We overlook the many stragglers by the wayside, stricken from the line of march by the strenuous efforts necessary to keep the step, and we fail to note the many already wearied to exhaustion, striving with the last atom of their vitality to maintain their place in line or to pass their file leader. The ever full and constantly enlarging almshouses, hospitals, insane asylums and penal institutions are eloquent testimonials of the pace that kills and the degeneration that must sooner or later tell the story of our past greatness to the future historian.

A brief study of our mortality rate will serve to establish a basis of camparison, and will assist us somewhat in arriving at a conclusion as to the exact status of our national vitality and efficiency. Our average duration of life has increased since 1879 from thirty-five to forty-five years, an increase of ten years in a little over a century. While these figures are based on rather doubtful returns, they are probably fairly correct, and will serve our purpose here very well. As a matter of comparison, the average duration of life in England is the same as ours, forty-five years, while India has the low average of only twenty-five years. A study of the mortality rate of the different countries of the civilized world shows a fairly constant dependency on the progress of medical science and the extent of public and personal hygiene practiced by their people. In Prussia, for instance, where these subjects are paramount, the average duration of life is increasing at the rate of twenty-seven years per century, while in India, where the reverse is said to be true, the average remains stationary at the lowest point. Our own increase is rated at fourteen years per century at the present time, a fair average, but far from that necessary if we would overcome the increasing handicap of an inexcusable morbidity and reach the goal of sixty years as our average duration of life, which we are told is possible. Flourens estimates that the normal life of mammals is five times their growing period. On that basis, man should normally live to the age of one hundred and fifty years. How near we may come to this mark depends largely on the progress of medical science and the practice of social and personal hygiene. As the specific cause and treatment of disease comes to light, and the principles of hygiene are developed and applied, both morbidity and mortality will decrease until it only remains to put up the bars against death and damage from violence to attain the maximum life allotted by our Creator to man.

We may not account for the increase in longevity by the assumption that we are physically a stronger people than ever before. Indeed, I believe the reverse to be true, and that we have ample causes for even a more rapid increase in longevity than we have actually experienced in the victories of medical science over plagues and pests, and the curtailment of life waste in other directions less conspicuous. In London, where statistics are available for many years back, we find the death rate reaching an average of eighty per one hundred thousand between the years 1680 to 1728, a period of pest. This rate has steadily decreased with the control of smallpox, cholera, and plagues and pests in general, and the better understanding of hygienic measures, until the death rate at this times does not exceed fifteen per one hundred thousand. In Prussia, according to well authenticated statistics, vaccination in two years reduced the death rate of smallpox from 24 to 1.5 per one hundred thousand. And it may be remarked, parenthetically, that the death rate from vaccination was *nil,* despite the crudeness of inoculation practiced at that time, a clear gain in human life of a few thousand per cent for the anti-vaccinationists to ponder over—if, indeed, they ponder at all. The vital statistics for Cuba and for the Isthmus of Panama had to be reconstructed from the ground up when those disease-ridden and pest-ridden countries fell into the hands of the United States army, with its dearly bought knowledge of the rôle played by the mosquito in the yellow fever plague of the tropics, together with the authority to enforce sanitary rules at liberty. As for our own country, we have positively eliminated yellow fever as a causative factor in our mortality, where in former days we had to accord it a prominent place. Thus we see the agencies which killed outright have been almost entirely subdued, while there has been hardly more than a perceptible impression made on the chronic, depleting, nerve-destroying diseases and practices. There can be no question but we are better off from the standpoint of morbidity and mortality than we have ever been before, but the end of betterment is in sight unless we take steps to overcome the insidious but less spectacular influences that are at work and have for some time been at work in the depletion of our national vitality.

Man possesses the highest organization of nerve force of all the animals, with corresponding potentiality and consequent responsibility. The supplanting of instinct by the development of reason removes the care of the human animal from nature to his own mental faculties, and he is capable of self-protection in proportion to the condition and development of his nerve force. As in the lower scale, the characteristics and traits of man are handed down from generation to generation, according to a law not altogether fixed, but well understood. The part played by environment in this process of hereditary transmission is important in itself, but much depends on the susceptible transmitted nature in the matter of the developmental effect of the impressions of environment. So the whole process may be termed hereditary, and heredity plays a most important rôle indeed in the conservation of national efficiency. Our supine indifference to this important subject is strange indeed, when we see on every hand the efforts made to meet these same conditions in the breeding of stock. The wise stock breeder protects his breeding animals from deterioration by surrounding them with proper sanitary conditions and protecting them from overwork, bad habits and other influences which might tend to reduce the value of the strain in transit from sire or dam to offspring. The vicious, degenerate or substandard in any respect is not permitted to breed, and often not allowed to be reared.

In heredity and environment, then, we have the mainspring of national vitality. Those influences which serve to build up the physical and mental strength of the individual react through posterity to the upbuilding of the race, and those conditions serving to weaken the individually physically or mentally are ultimately productive of deterioration and degeneracy.

Any influence whatsoever acting in a way to deplete nerve force weakens the individual. Perception is made possible through the development of the nerve cells of the brain, and an abnormal development, or a depleting process, tends to render perception abnormal. Through the sense of perception alone is the individual able to conduct himself normally. The natural expectation of society is that every member thereof conduct himself along well-established lines, and any departure from this rule tends to chaos and disorganization. Deviations from the normal conduct of man, except such as occur under the influence of excitement or the toxic effect of alcohol or drugs, is a sign of degeneracy, and ranges in degree from the neurasthenic to the maniac.

From the standpoint of national efficiency, the physically weak, though not a degenerate in the ordinary acceptance of the term, must be considered, and aside from his tendency to beget real degenerates. A weakened constitution, either from lack of development or from disease or dissipation, is not only likely to beget substandard posterity, but is not productive of its share of the common weal, and is peculiarly liable to the ills of nature and the temptations of man. Inherently, and by precept and example, then, he becomes a menace to his associates. This type, for the purposes of discussion, may be merged with the actual degenerate or pervert, and the whole considered as the deteriorating element. The cause of degeneracy and national deterioration, then, are those conditions which serve to weaken the nerve force of our people and render them incapable of a normal understanding and a normal incentive to right living. These causes are many, too many to give them all full consideration in a single discussion, but they run in groups through a few special conditions, in which manner I choose to consider them.

We may well begin with the dilution of our national vitality by the hordes of substandard aliens annually admitted into our country from the older countries of Europe and Asia, where degeneration has progressed for centuries under extreme conditions of poverty, disease and oppression. The living conditions and habits of thought and action of these people are reproduced in this country as nearly as possible, and the influence of their lives and of their personal conduct upon our own poorer classes is of even greater importance than the direct results of their mere presence. The government presumes to restrict immigration to the exclusion of the diseased, the criminal or the pervert, but it is out of the question to expect that any practical system of inspection would be successful in detecting any but the very patent cases, and for every one detected and denied admission thousands are admitted whom we would do well to exclude. Depravity, degeneracy and many of the constitutional diseases so disastrous to society may not always be detected, and more often than otherwise really serious cases pass our portals of entry with ease. That many of these people are incapable of realizing their responsibilities as members of society and as citizens must be conceded. That a large percentage are fundamentally weak, vicious, and criminally inclined, rotten with venereal diseases and depraved in habits, will also not be denied. If the continued influx of this class did not itself keep up the supply, we could count on their disposition to procreate, all out of proportion to that of the higher classes, to prevent their entire elimination as a dangerous factor.

Closely allied to this source of degeneration is that of our own poorer classes, differing only in that they have not been exposed for as many generations to such unfavorable conditions. We find them a national menace primarily in the congested cities of the first class, where they are herded—men, women and chidren—into unsanitary tenement districts, with little or no opportunity for fresh air, wholesome exercise or influences of any kind for their elevation in the social scale. Their poverty denies them proper food and proper raiment, and an illy-nourished body, unprotected from the rigors of climate and with an insufficient supply of life-giving oxygen, can be productive only of a substandard constitution. Add to these unfavorable conditions long, exhaustive hours of labor, too often as unsanitary as their living conditions, and often including practically every member of a family, and degeneration is made certain. The natural inclination of the nerve-depleted human is to seek stimulation, or some satisfaction for a craving inherent in the condition, and dissipation as varied as opportunity offers is the result. Thus we find that drink and drugs, and the immoral practices suggested in the brothel and the public dance halls, abound among these unfortunate people. That tuberculosis, syphilis and gonorrhea should find them an easy prey is not strange, and that the children of such people should show all the stigmata of degeneration is to be expected. The children of the slums are notoriously inclined to depravity and crime, and a large proportion ultimately end their days in the almshouse, insane asylum or penitentiary.

Not alone among the poorer classes do we find potent factors in degeneracy. Our very wealthiest and highest classes of citizens are exposed to dangers none the less powerful, though perhaps less evident. The strain incident to modern competition produces a nerve depletion equally as dangerous as that acquired in any other way. It is accompanied by the same craving, the satisfaction of which results in dissipation, and inclines to the production of the same vicious cycle found in the lower classes. The wealth and the opportunity of this class for indulgence serves to match in ultimate results the degeneracy of the lower classes. The rules of polite society and the opportunities for care and treatment offered by their wealth alone serves to keep the standard a bit elevated. The unemployed among the opulent classes, by virtue of the time they have to spare, are even more ready victims to the wiles of dissipation than their overworked companions, and therefore yield a richer harvest of perverts. Our private sanitariums, insane asylums and even penal institutions offer a plentiful supply of degenerates, both acquired and hereditary, recruited from the ranks of our very best society.

Aside from the influence of class and environment as a factor in the production of degeneracy, we must consider the effect of disease as an independent agent. Among the diseases entitled to special consideration, because of their chronic depleting influences, tuberculosis, syphilis and gonorrhea chiefly concern us. The acute diseases, even the minor ailments, are of importance, but mainly because of their predisposing influence in the production of the more important chronic constitutional diseases. The very common picture of the cold, then grippe or pneumonia and finally tuberculosis serves to illustrate the rôle played by these minor and more important acute diseases. Other examples of this succession of events may be called to mind by anyone familiar with sickness to any extent at all. It is estimated by good authority that there are three million people sick all the time in this country, and that an average loss of time of thirteen days per year is thereby incurred.

The nerve depletion incident to chronic diseases plays the same rôle in the production of degeneracy that it does from other causes, less, perhaps, the average vicious example and personal influence of the depraved from dissipation. Here, as elsewhere, the laws of heredity serve as the principal promoter of deterioration, and unrestricted personal freedom serves to extend the process in its own generation by conveying the disease to others, and by the adoption and practice of vicious habits induced by the physical weakness of the sick.

There are from three hundred thousand to five hundred thousand people constantly sick of tuberculosis in this country, and it is calculated that eight million people today alive are destined to die before their time of this preventable disease. Fully half of those sick of this disease are incapacitated physically, and when it is considered that many of them are without any knowledge whatsoever of the dangers to posterity through the agency of heredity, or to immediate generations by contact, the significance of the situation is evident. The liability of the ignorant tuberculous to yield to the murderous importunities of the patent medicine men, the erroneous impression that alcohol is a cure for consumption and the abnormal craving of the depleted system, all conspire to make of these sick people alcohol and drug habitues—all of which accentuates the seriousness of hereditary taint.

Of the thousands of defectives in the United States, by far the greatest number directly attributable to anyone cause are due to the venereal diseases. I do not except alcohol or drug addictions, as I believe these habits are more often than otherwise due to defective nerve force incident to other factors. In gathering our statistics on the subject of drink and drugs, we too often fail to given due consideration to this feature of the case, but alienists and sociologists are realizing and testifying to this end more and more as the investigations go on. It is unfortunate that we have no dependable statistics on the prevalence of venereal diseases, but the private nature of the diseases, and the ordinary method of their transmission, renders it impossible to secure provision for their registration in public health and vital statistic laws at the present time. Those who have investigated the subject tell us, however, that there are two million syphilitics in the United States, and that of the eight hundred thousand men reaching maturity annually from 75 to 80 per cent will have contracted gonorrhea before the age of 30. One quotation from the statistics of the army will serve to reinforce these estimates.

In 1904 the troops in the Philippines suffered from venereal diseases four times the morbidity of any other one disease. Of every one thousand soldiers, two hundred and ninety seven suffered from either syphilis or gonorrhea, and twenty-two out of every one thousand were constantly infected from one or the other of these preventable diseases. It may be true that the fact that the soldier is, as a rule, unmarried and of the age most likely to seek sexual gratification, renders him peculiarly liable to contract venereal diseases. Still, it is not believed that conditions in this respect are any better in civil life. The immediate effect of venereal diseases in the production of the lame, the halt and the blind, the neurotic, psychotic and the insance is appalling, but the ultimate effect through heredity passes comprehension. The direct and the by-effects of syphilis and gonorrhea are well known and hardly require further discussion. I would call attention, however, to the deteriorating influence on our national integrity of the separated families, sterile homes, invalided innocent women, and substandard offspring, in thousands of instances where the true cause is unsuspected, but dependent on the venereal peril.

The remedy for deterioration and degeneracy is beyond the scope of this discussion, but I venture to suggest that the medical profession could plan a system of control, which, applied by adequate laws supported by adequate funds, would result in placing the human animal in position to breed himself up to the highest possible standard in a comparatively few generations. The State very laudably provides for the care and protection of the degenerate, but takes no steps to curtail the source of supply. This looks like folly of the most inexcusable sort, but there is a measure of defense for this attitude in the lack of understanding of such matter on the part of the laity, and it is our province and our duty to remove this prime obstacle in the way by giving publicity to these questions, that the people may be educated to a full sense of realization of the situation. Perhaps then we may have a system of public health laws, adequate in extent and application, and brought into harmonious relation through the State authorities by a National Department of Health. We may then see the propagation of degenerates made impossible by humane sterilization, and may even see the last of the triple arch enemies of mankind, syphilis, gonorrhea and tuberculosis, as we have seen the practical disappearance of yellow fever and smallpox, under only a fair amount of attention by health authorities. We may then see the one hundred and eighty thousand cases of insanity at present in our public asylums dwindle in number in the same ratio that it is today increasing, and our people will wonder that their most precious interests had been so long neglected because of the mistaken sentiment that personal liberty should not be made subservient to the common welfare of society.

